# Enhancing autonomous vehicle navigation using SVM-based multi-target detection with photonic radar in complex traffic scenarios

**DOI:** 10.1038/s41598-024-66850-z

**Published:** 2024-07-28

**Authors:** Sushank Chaudhary, Abhishek Sharma, Sunita Khichar, Yahui Meng, Jyoteesh Malhotra

**Affiliations:** 1 School of Computer, Guangdong University of Petrochemical Technology, Maoming, 525000 China; 2 Department of Electronics Technology, Guru Nanak Dev University, Amritsar, India; 3 Department of Electrical Engineering, Chulalongkorn University, Bangkok, Thailand; 4 School of Science, Guangdong University of Petrochemical Technology, Maoming, 525000 China; 5 Department of Electronics and Communication Engineering, National Institute of Technology, New Delhi, India

**Keywords:** Smart cities, Autonomous vehicles, ITS, SVM, Photonic radar, FMCW, Resolution, Lasers, LEDs and light sources, Optical techniques, Other photonics

## Abstract

Efficient transportation systems are essential for the development of smart cities. Autonomous vehicles and Intelligent Transportation Systems (ITS) are crucial components of such systems, contributing to safe, reliable, and sustainable transportation. They can reduce traffic congestion, improve traffic flow, and enhance road safety, thereby making urban transportation more efficient and environmentally friendly. We present an innovative combination of photonic radar technology and Support Vector Machine classification, aimed at improving multi-target detection in complex traffic scenarios. Central to our approach is the Frequency-Modulated Continuous-Wave photonic radar, augmented with spatial multiplexing, enabling the identification of multiple targets in various environmental conditions, including challenging weather. Notably, our system achieves an impressive range resolution of 7 cm, even under adverse weather conditions, utilizing an operating bandwidth of 4 GHz. This feature is particularly crucial for precise detection and classification in dynamic traffic environments. The radar system's low power requirement and compact design enhance its suitability for deployment in autonomous vehicles. Through comprehensive numerical simulations, our system demonstrated its capability to accurately detect targets at varying distances and movement states, achieving classification accuracies of 75% for stationary and 33% for moving targets. This research substantially contributes to ITS by offering a sophisticated solution for obstacle detection and classification, thereby improving the safety and efficiency of autonomous vehicles navigating through urban environments.

## Introduction

Evolution of smart cities has brought about many improvements in the quality of life for people living in urban areas. Transportation is indeed a backbone of every city, and the development of intelligent transportation systems (ITS) has greatly enhanced the performance of transportation systems^1^. Connected and autonomous vehicles, in particular, have the potential to greatly improve travel safety, traffic management, environmental impact, and the use of transportation for both commercial users and the general public^2^. The integration of various sensing functions into a single transceiver platform can greatly benefit the development of future intelligent vehicles. This can reduce costs and improve efficiency and reliability, as well as simplify the overall system^3^. By working autonomously and cooperatively, intelligent vehicles can greatly improve the performance of transportation systems by exchanging information on traffic, road, and weather conditions to adjust speed and route and avoid congestion and potential hazards, leading to reduced travel time, fuel consumption, and emissions^4^. Self-driving cars are equipped with different types of sensors such as radar, lidar, cameras, and ultrasonic sensors, which help them understand their environment and make choices^5^. Radar sensors are commonly used in autonomous vehicles to detect objects and obstacles, especially in poor weather conditions or low light situations where other sensors may not be as effective^6^. Conventional electrical radars have limited frequency and bandwidth when using purely electrical techniques. While it's possible to boost the bandwidth using several stages of frequency conversion, the efficiency of both the transmitted signal and the analog-to-digital converters (ADCs) tends to drop quickly as the operating bandwidth gets wider^7,8^. This leads to restricted resolution and processing speed for the radar. To overcome these limitations, microwave based photonic radar schemes have been proposed by many researchers^9,10^. Photonics devices, known for their ability to work at high frequencies and over a wide range of bandwidths, can be used to increase the frequency and bandwidth of radar systems. This makes microwave photonic technology a strong candidate for improving radar capabilities. There have been several proposals and successful demonstrations of broadband radars based on photonics, highlighting the practicality and benefits of using this technology^11–14^. Photonics-based broadband radars have proven to be particularly effective in achieving ultra-high range resolution, with demonstrated capabilities of several millimetres^15^. In 2014^16^, P. Ghelfi et al. showcased the first microwave photonic radar that utilized a mode-locked laser for creating signals and optical sampling. Yet, its operating bandwidth and the ability to measure distances accurately were limited due to a low pulse-repetition frequency. To overcome these limitations, various types of broadband linear frequency-modulated (LFM) signal generators have been employed in microwave-based photonic radar systems. These include techniques like converting optical wavelength to frequency and using photonic methods for digital-to-analog conversion^17,18^. Many broadband microwave-based photonic radar systems typically utilize microwave photonic frequency multiplication for generating signals in the transmitter. Additionally, they employ microwave photonic frequency mixing for broadband photonic de-chirping in the receiver^19,20^. The main contributions in the current work can be highlighted as follows:

- *Integration of Photonic Radar Technology* Introduction of a novel FMCW photonic radar system that enhances multi-target detection capabilities in complex traffic scenarios.
- *High Range Resolution* Achieving an impressive range resolution of 7 cm using an operating bandwidth of 4 GHz, even under adverse weather conditions.
- *Low Power and Compact Design* Development of a radar system with low power requirements and a compact design suitable for deployment in autonomous vehicles.
- *Accurate Target Detection* Demonstrating through numerical simulations the system's ability to accurately detect stationary and moving targets at varying distances and movement states.
- *Support Vector Machine (SVM) Classification* Utilization of SVM classification to improve target detection and classification accuracy, achieving 75% accuracy for stationary targets and 33% for moving targets.
- *Enhanced Safety for Autonomous Vehicles* Providing a sophisticated solution for obstacle detection and classification, thereby improving the safety and efficiency of autonomous vehicles navigating through urban environments.

## Related works

Some key works on LFM based photonic based microwave radars has been discussed. In 2021^21^ a photonics-based technique was introduced for creating multiple LFM signals. This method used a dual-parallel Mach–Zehnder modulator (DP-MZM) along with a dual-band LFM signal. This approach successfully generated eight LFM signals simultaneously, reaching up to 27 GHz. It also offered the advantage of multiplied bandwidth and enhanced pulse compression capability. An experiment is conducted to demonstrate the approach, and the performance of the multi-band LFM signal is verified. Another study in 2021^22^ presented a photonics-based system that uses an acousto-optic modulator for simultaneous measurement of angle of arrival (AOA) and frequency. The system uses an optical frequency shift loop and Van Cittert-Zernike theorem to suppress undesired frequency components and estimate AOA from multiple RF sources. The proof-of-concept experiment demonstrated successful measurement of multiple targets with different frequencies and distinguishing incoherent microwave sources in the angular direction. A review article in 2021^14^ discusses the advantages and potential applications of microwave photonic (MWP) radar in areas such as intelligent autonomous systems and cyber-physical systems. It provides an overview of recent advancements and future directions in MWP radar, covering various components, design challenges, and issues. A comparative study of different MWP radar applications is presented, along with a discussion of possible future research directions.

In 2022^7^, a new microwave photonic radar approach was introduced by an author, characterized by its generation and processing of broadband linear frequency-modulated signals without relying on RF (Radio Frequency) sources. This entirely photonics-based radar system is notable for its high reconfigurability, affordability, and simple design. It exhibits superior capabilities in range and 2D imaging resolutions. This development holds promise for future radars that are multifunctional, adaptive, and more compact in size. Another author in 2022^23^ proposed a a photonics-based Time Division Multiplexing (TDM) Multiple-Input and Multiple-Output (MIMO) radar system. This system uses microwave photonic frequency octupling combined with optical TDM techniques to achieve high-resolution imaging. They developed a 4 × 8 TDM-MIMO radar with each transmission channel operating at a bandwidth of 8 GHz. This setup achieved a range resolution of 1.9 cm and an angular resolution of 1.1°. Additionally, a broadband back projection imaging algorithm with phase compensation was introduced, aimed at providing high-resolution imaging of both single and complex targets. Another article in 2022^24^ demonstrated a technique for high-resolution imaging using a microwave photonic array radar. This system incorporated a novel scanning time delay-compensated digital beam forming method. It utilized photonic frequency quadrupling and de-chirping techniques for the generation and processing of signals. An experimental setup of a 1 × 16 array radar, operating with an 8 GHz bandwidth, was demonstrated. This setup successfully achieved high-resolution imaging of near-field targets, along with precise beam correction and effective suppression of grating lobes. In 2023^25^ a study presented a photonic integrated sensing and communication (ISAC) system that combines wireless communication with multi-target detection capability. The system uses optical methods to enable accurate range and Doppler measurements and a frequency-domain method to enhance resolution and real-time processing capability while reducing computational requirements.

## Applications of machine learning in autonomous vehicles

The progress in Machine Learning has ushered in novel prospects for the automotive sensing industry to transition into Industry 4.0^26^. Using Reinforcement Learning (RL) techniques such as Q-Learning, Deep Q Network (DQN), and Deep Deterministic Policy Gradient (DDPG) optimizes offloading decisions in UAV-assisted MEC, improving energy efficiency and reducing service delays. Additionally, Artificial Neural Networks (ANNs) accurately predict signal strength despite environmental impacts, ensuring reliable data transmission and efficient resource management^27,28^. Machine Learning algorithms possess the capability to scrutinize historical and present data to forecast forthcoming conditions. The progress in machine learning has significantly advanced the automotive sensing industry, enabling the development of predictive models for autonomous vehicle behavior^29^. Support Vector Machines (SVMs) are particularly effective in this context, as they can classify and detect targets by analyzing unique spectral signatures. By projecting data into higher-dimensional spaces, SVMs improve target detection and classification, even in cluttered or noisy environments. Their applications range from handwritten digit recognition to object recognition, demonstrating efficacy in both academic and industrial setting. Recent advancements in the application of machine learning to autonomous vehicles are explored in this section. In 2020^30^ a study discussed the application of machine learning algorithms in autonomous driving systems for tasks such as motion planning, vehicle localization, and fault diagnosis. The algorithms are compared based on metrics such as mean intersect in over union and false positives per image. The study contributes to the understanding of machine learning in autonomous dring systems. Another article in 2020^31^ introduced a traffic control system based on machine learning designed to enhance the efficiency of Autonomous Vehicle Systems (AVSs) used in semiconductor manufacturing. This system is capable of predicting traffic congestion in critical bottleneck areas and dynamically selects routes for the AVSs that are less congested. Through experimental evaluation and simulation studies, it was demonstrated that this new approach surpasses the current methods in terms of delivery time, transfer time, and queuing time. These results underscore the significant role machine learning can play in boosting the performance of AVSs, while simultaneously reducing the need for human monitoring and control. In 2021^32^ a study provides an overview of the use of machine learning and deep learning algorithms in various tasks of autonomous driving systems, including motion planning, vehicle localization, pedestrian detection, traffic sign detection, road-marking detection, automated parking, vehicle cyber-security, and system fault diagnosis. The algorithms are compared based on metrics such as mIoU, AP, missed detection rate, FPPI, and false frame detection. The study contributes to a comprehensive review of these algorithms for autonomous driving systems. Another article in 2021^33^ discusses a model-based support vector machine (SVM) approach for steering actuator fault diagnosis in automated vehicles. The system model generates the residual signal as training data for the SVM classification algorithm to diagnose faults. To improve the classification performance, undersampling with linear discriminant analysis and threshold adjustment using grey wolf optimizer are proposed. The algorithm shows superiority in classification compared to existing methods, and experimental results demonstrate its effectiveness in steering actuator fault diagnosis. In 2022^34^ researchers proposed an imitation learning-based decision-making framework to provide recommendations for entering roundabouts in a driving assistance system. The framework uses deep policy networks and observations from a monocular camera to determine the best timing for entry. The approach outperforms state-of-the-art supervised learning methods, demonstrating its potential in enhancing driving safety. A survey in 2023^35^ explores the challenges of achieving accurate environment context for effective decision-making in autonomous and connected vehicles using a heterogeneous set of onboard sensors and V2X communication. It highlights the influence of data pre-processing and fusion, along with situation awareness, toward optimal execution of critical maneuvers. The survey provides potential research directions to address the major hiccups and achieve accurate contextual awareness. Another study in 2023^26^ provides an overview of different sensor modalities and associated data processing techniques used for automotive perception, which involves understanding the external driving environment and internal state of the vehicle. The role of machine learning, validation methodologies, and safety aspects are also discussed. The technical challenges for each aspect are analyzed, and future research opportunities for wider deployment are outlined.

In this work, we have proposed FMCW based photonic radar in coherent detection configuration in heterodyne formation. The FMCW-based photonic radar system is well-suited for autonomous cars and can provide accurate and reliable measurements of target characteristics. The proposed system is equipped with spatial laser to detect and range 4 targets under complex traffic scenario. The Doppler Effect has been considered and system is also tested for the adverse weather conditions. The system is also tested for collision time in moving conditions. Lastly the proposed system is trained using SVM model for classification of targets under different circumstances.

## System modelling and working principle

**Figure 1:**
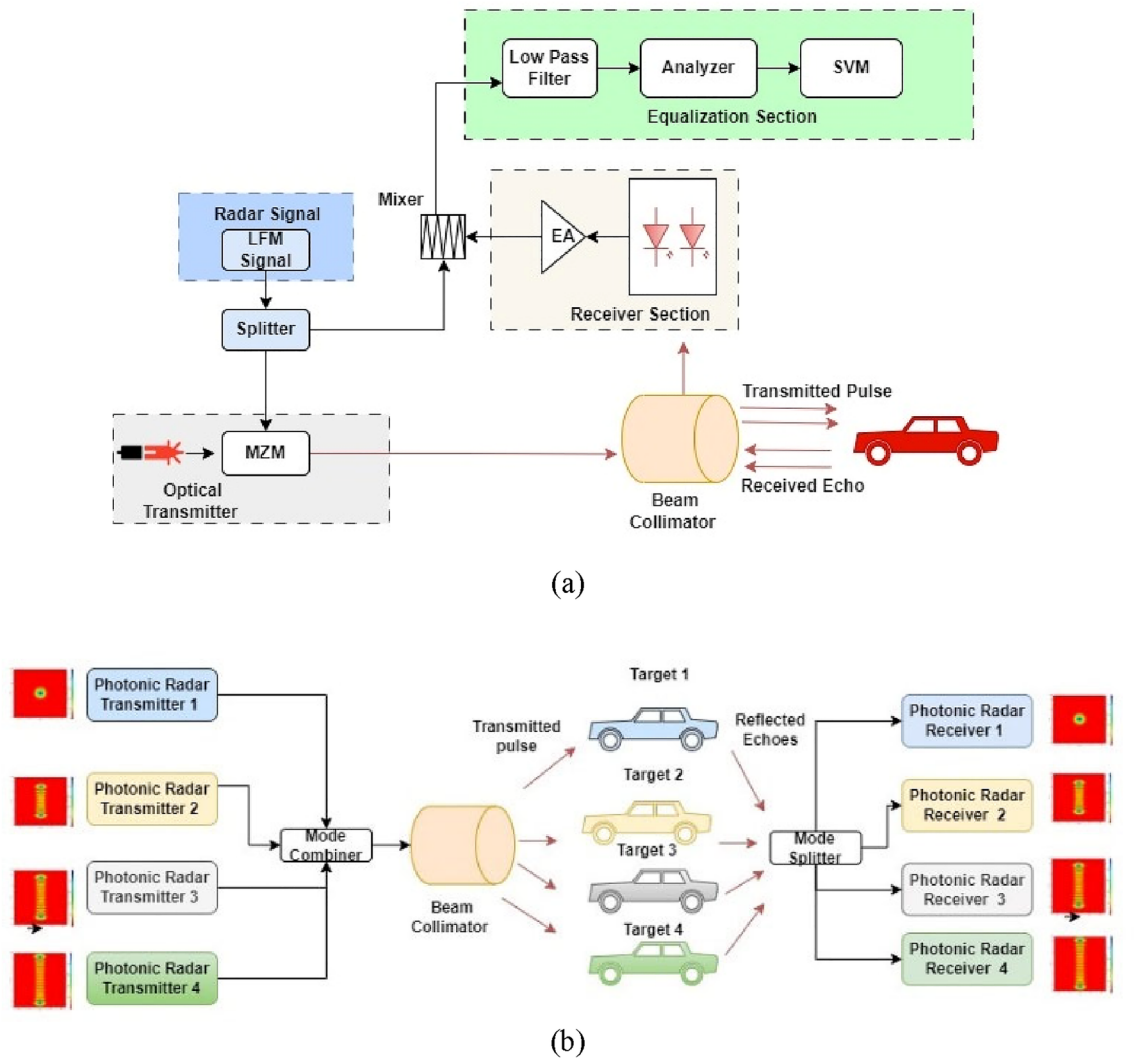
Proposed SVM-MDM photonic radar (**a**) Transmitter and Receiver (**b**) MDM concept.

The proposed photonic radar system in coherent detection topology, as shown in the Fig. 1 below, uses FMCW technique and is designed to meet the compact size and low input power requirements of autonomous cars. The proposed photonic radar works by using the Doppler Effect. This means it copies the signal it sends out, but the signal it gets back is slightly delayed.

The system employs a linear sweep of the frequency band to estimate the time delay and Doppler shift information. This information is crucial for measuring the range (distance) and velocity of the target object. This way, the system can extract valuable information about the target's movement and location. To enhance the decoupling effect between speed and range in the system^36^, a waveform generator is utilized, which produces a triangular sweep waveform with LFM^37^. The waveform generator is configured with a start frequency of 77 GHz and a bandwidth of 4 GHz. This triangular sweep waveform with LFM serves as the transmitted signal in the system. This waveform enables the system to achieve more precise and accurate measurements by reducing interference between the speed and range signals.

**Figure 2:**
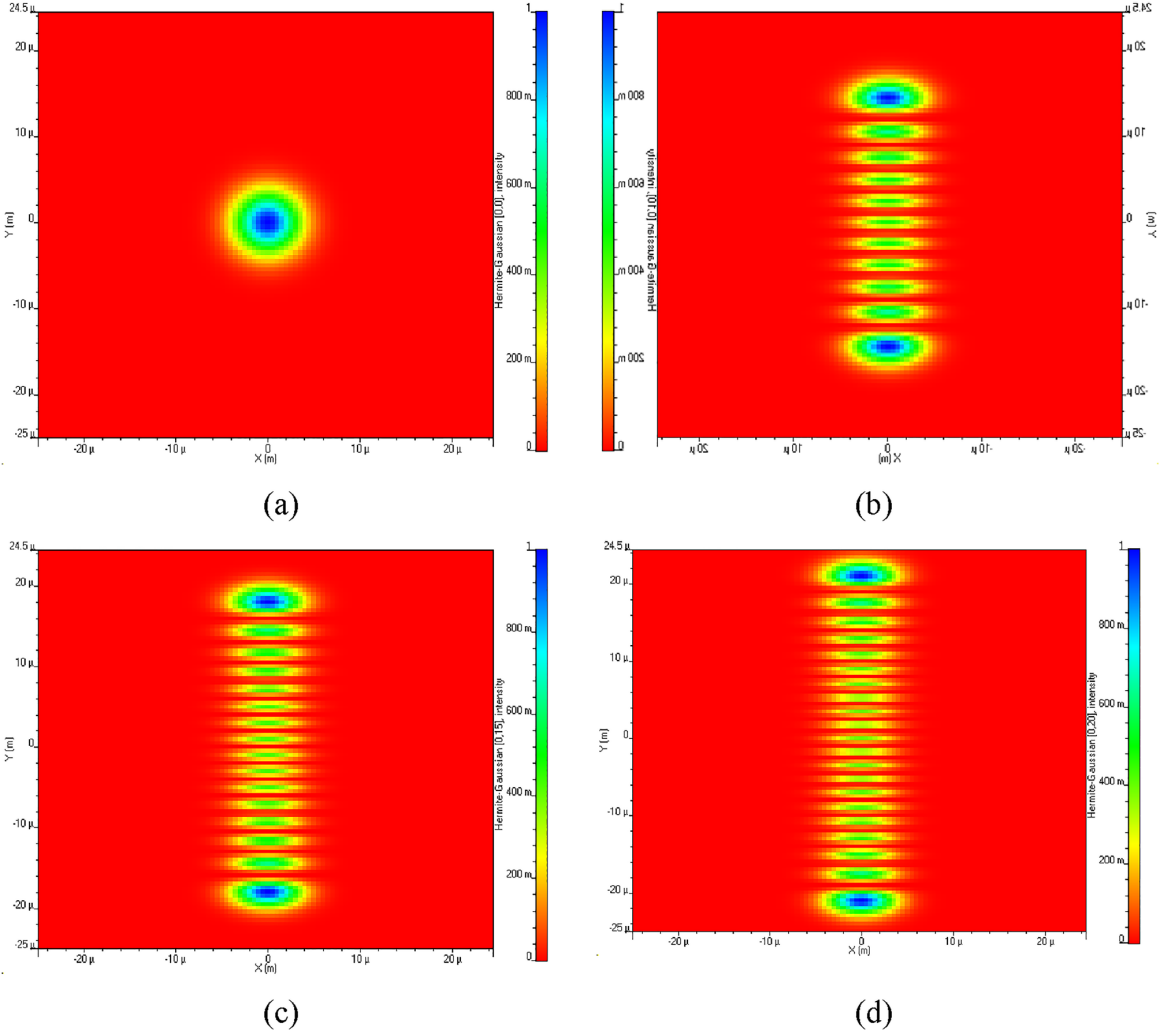
Hermite-Gaussian mode profile (**a**) HG 0 0, (**b**) HG 0 10, (**c**) HG 0 15 and HG 0 20.

Once the LFM signal is made, it's mixed with a light signal using a special device called a DP-MZM. This is done with the help of a laser that's always on (continuous-wave laser). After this laser, we use another device called a mode generator to create Hermite-Gaussian (HG) modes. HG modes are used to carry the signal over a single wavelength, which enables the system to achieve higher spectral efficiency and better signal quality. HG modes are a set of orthogonal modes that can be used to describe the spatial distribution of electromagnetic fields in a resonant cavity. HG modes have a Gaussian amplitude profile and a specific phase distribution, which makes them useful for carrying optical signals in optical communications systems. By generating HG modes, the system can effectively encode information onto the optical carrier signal and transmit it over the free-space channel with high efficiency and minimal interference. Four HG modes used to carry the signal for detection of four individual targets are as shown in Fig. 2.

The modulated signal is then transmitted over a free-space channel using a telescope. In order to achieve coherent detection, the differential pair DP-MZM is operated in a null stage. The DP-MZM is set up with a switch voltage of 4 V and plus 1 and minus 1 V as bias voltages at both of its inputs. This configuration helps ensure that the DP-MZM operates in the desired linear range, facilitating accurate and coherent detection in the system. The output signal that comes from the DP-MZM modulator is called E(t) in Eq. (1) ^38^.

1 $$ E(t) = \frac{{E_{in} (t)}}{{10^{IL/20} }} \times \left( {\begin{array}{*{20}l} {(\gamma \times e^{{{{(j\pi v_{2} (t)} \mathord{\left/ {\vphantom {{(j\pi v_{2} (t)} {V_{\pi RF} + {{j\pi v_{bias2} } \mathord{\left/ {\vphantom {{j\pi v_{bias2} } {V_{\pi DC} }}} \right. \kern-0pt} {V_{\pi DC} }}}}} \right. \kern-0pt} {V_{\pi RF} + {{j\pi v_{bias2} } \mathord{\left/ {\vphantom {{j\pi v_{bias2} } {V_{\pi DC} }}} \right. \kern-0pt} {V_{\pi DC} }}}}}} )} \hfill \\ { + ((1 - \gamma ) \times e^{{{{(j\pi v_{1} (t)} \mathord{\left/ {\vphantom {{(j\pi v_{1} (t)} {V_{\pi RF} + {{j\pi v_{bias1} } \mathord{\left/ {\vphantom {{j\pi v_{bias1} } {V_{\pi DC} }}} \right. \kern-0pt} {V_{\pi DC} }}}}} \right. \kern-0pt} {V_{\pi RF} + {{j\pi v_{bias1} } \mathord{\left/ {\vphantom {{j\pi v_{bias1} } {V_{\pi DC} }}} \right. \kern-0pt} {V_{\pi DC} }}}}}} )} \hfill \\ \end{array} } \right) $$

where *E*_*in*_*(t)* is input signal to the laser source, and the insertion loss of the system is represented by *IL*. The two input bias voltages applied to the lower and upper arms of the Mach–Zehnder modulator (MZM) are denoted as '*v*_*1*_' and '*v*_*2*_', respectively. Once the modulated signals are generated by each transmitter carrying different modes, they are combined using a combiner. Once the combined signal is boosted using an optical amplifier, which has a gain of 10 decibels (dB) and a noise figure of 4 dB, the amplified signal is then sent over a free-space channel. This transmission is facilitated using a telescope lens to focus and direct the signal. For modeling Free Space Optics (FSO), we use a Gamma Gamma fading channel is shown in Eq. (2). This model allows for the characterization of the channel's behavior, including the effects of atmospheric turbulence and fading, which can impact the signal quality and reliability during transmission through the free-space medium. This channel model takes into account the various factors that can affect the transmission of the optical signal, such as atmospheric turbulence and scintillation^39^.

2 $$ P_{Received} = P_{Transmitted} \times \frac{{d_{R}^{2} }}{{\left( {d_{T} + \theta R} \right)^{2} }}10^{{ - \alpha \frac{R}{10}}} $$

The transmitter and receiver aperture diameters are denoted by *d*_*T*_ and *d*_*R*_, respectively. The atmospheric turbulence effects are represented by the variable *α*.

The distance between the sender and the receiver is called *R*, and the spread of the beam is shown by *θ*. At the receiver, a special light-sensitive device called a photodiode is used to receive the optical signal, and it's set up in a balanced way for detection. This configuration involves mixing the laser signal with the echo signal at the receiver, which allows for improved sensitivity and lower noise levels. The strength of the reflected echo signal from the target, denoted as *P*_*r*_, is given by Eq. (3) ^38^.

3 $$ P_{r } = \left\{ {\begin{array}{*{20}l} {P_{t} \frac{{\rho_{t } D^{2} \tau_{opt} \tau_{atm}^{2} }}{{4R^{2} }}} \hfill & {for \;extended\; target} \hfill \\ {P_{t } \frac{{\rho_{t } A_{t} D^{2} \tau_{opt} \tau_{atm}^{2} }}{{4R^{2} A_{ill} }}} \hfill & {for \;any\; target} \hfill \\ \end{array} } \right. $$

where, the receiver's aperture diameter is denoted by *D*. The target area is represented by *A*_*t*_ indicating the size of the target being observed. The reflectivity of the target is denoted by *ρ*_*t*_, which indicates the efficiency with which the target reflects the incident signal. The transmission loss of the system is given by *τ*_*opt*_, while the atmospheric loss is denoted by *τ*_*atm*_. The optical-to-electrical conversion at the receiver is performed using two photodiodes, denoted as *P*_*D1*_ and *P*_*D2*_. The optical electrical field at the first photodiode, *E*_*pd1*_, and the second photodiode, *E*_*pd2*_, are represented by Eqs. (4 and 5), respectively.

4 $$ E_{pd1} = \frac{1}{\sqrt 2 }\left[ {E_{lo} \left( t \right) + jE_{ref} \left( t \right)} \right] $$

5 $$ E_{pd2} = \frac{1}{\sqrt 2 }\left[ {E_{lo} \left( t \right) + E_{ref} \left( t \right)} \right] $$

The local oscillator electrical field, denoted as *E*_*lo*_*(t)*, is represented by Eq. (6):

6 $$ E_{lo} \left( t \right) = \sqrt {P_{lo} } e^{{j\left( {\omega_{o} \left( t \right) + \theta_{o} \left( t \right)} \right)}} $$

To get the beat signal, the LFM (Linear Frequency Modulation) signal created at the transmitter is mixed with the signal detected by the photodiode. This combined signal is then passed through a rectangular low-pass filter. The purpose of the low-pass filter is to remove any high-frequency noise or unwanted components from the signal, allowing only the desired beat signal to pass through for further processing and analysis. The resulting beat signal, denoted as *S*_*b*_*(t)*, is represented by Eq. (7) ^38^:

7 $$ S_{b} \left( t \right) = \Re \times A_{lo} \times \sqrt {P_{lo} \times P_{r} } \cos \left[ { 2\pi f_{start} \tau + \frac{\pi \beta }{{T_{m} }} \left( \tau \right)^{2} + 2\pi f_{r} \left( t \right)} \right]\sin \left[ {\omega_{d} \left( t \right) + (\theta_{o\left( t \right)} - \theta_{lo\left( t \right)} )} \right] $$

The range frequency *f*_*R*_ of the beat signal is expressed mathematically as:

8 $$ f_{r} = \frac{2 \times R \times B}{{T_{m } \times C}} $$

where target range from the photonic radar equipped vehicle is given as *R*, system bandwidth is given by *B*, *T*_*m*_ is the time taken by transmitted signal to sweep across a range of frequencies and speed of light as *c*.

## System modelling and working principle

**Figure 3:**
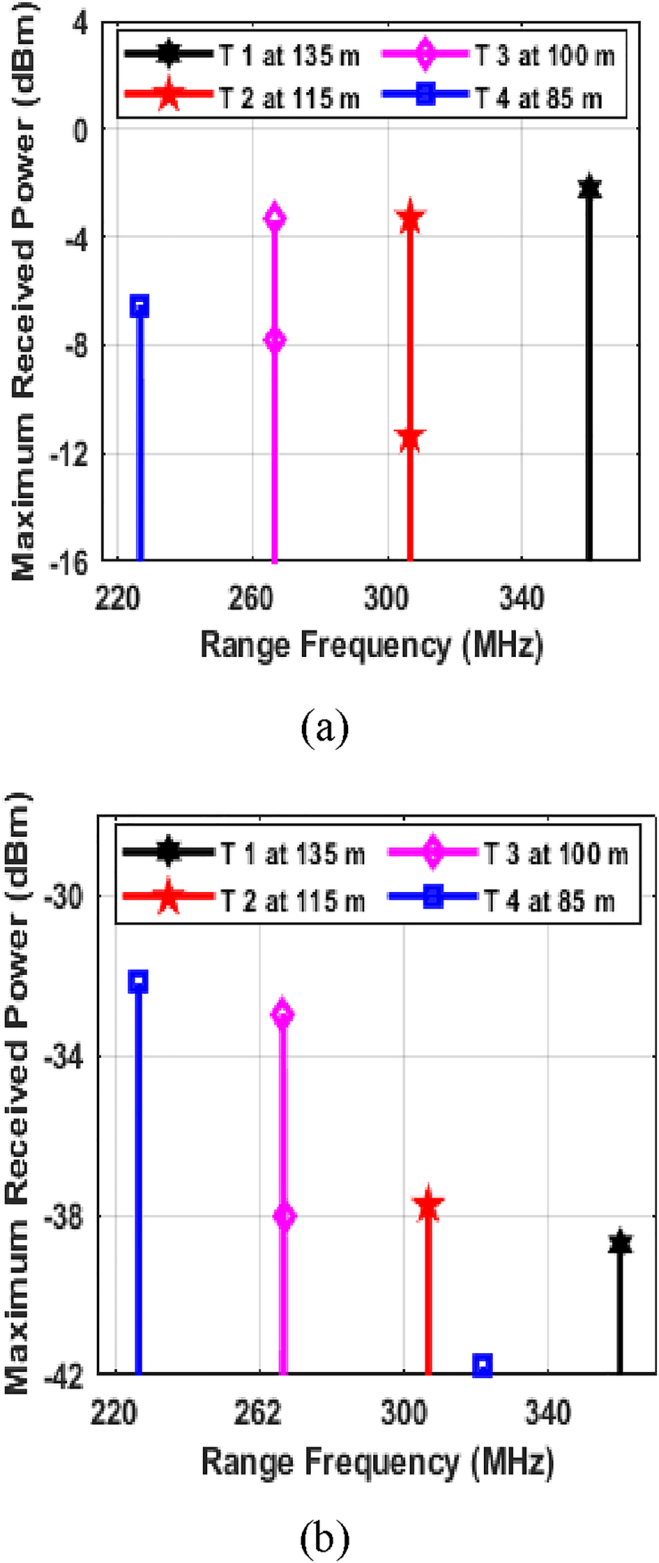
Stationary target detection (**a**) at 0.2 dB/km Clear weather conditions and (**b**) at 297.38 dB/km dense fog conditions; T represents target and m represents meters.

The numerical simulation-based results of proposed system are presented and discussed in this section. First the working of Photonic radar is analysed under different conditions. In Fig. 3, the proposed system is tested for detection of stationary targets.

As shown in Fig. 3a, the photonic radar system we designed was able to detect all four separate targets when the weather was clear. The first target was placed 135 m away from the system, the second target was 115 m away, the third was 100 m away, and the fourth was 85 m away. The range frequencies from the first target were observed at 360 MHz, whereas the second target showed echoes at 306.6 MHz. In a similar pattern, the third target's echoes were noted at 266.66 MHz, and the fourth target's at 226.66 MHz. These empirical observations were then cross-referenced with theoretical calculations derived from Eq. (8). The congruence in the results validated the precision and reliability of our proposed system in detecting all targets accurately, underscoring its effectiveness in practical applications. Additionally, the system was tested under various atmospheric turbulences, as shown in Fig. 3b, to evaluate its robustness and reliability in different environmental conditions. The performance of photonic radars in the microwave band is affected by atmospheric disorders, especially rainfall and fog. Rain affects optical signals less than fog because raindrops are smaller. We use the Kim model to describe rain's impact and the Mie scattering empirical model for fog. The Marshall Palmer distribution calculates rain's power law variables, while the International Telecommunication Union's (ITU)^40^ visibility code measures rain and fog attenuation. This code indicates 0.2 dB/km for clear weather, up to 75 dB/km for heavy fog, and 297.38 dB/km for dense fog. Figure 3b shows that despite atmospheric turbulence and dense fog, our system can detect targets with good maximum received power. We found the same range frequency for target detection under clear and foggy conditions, proving the radar's effectiveness in dense fog. However, more atmospheric disturbance leads to less echo power.

**Figure 4:**
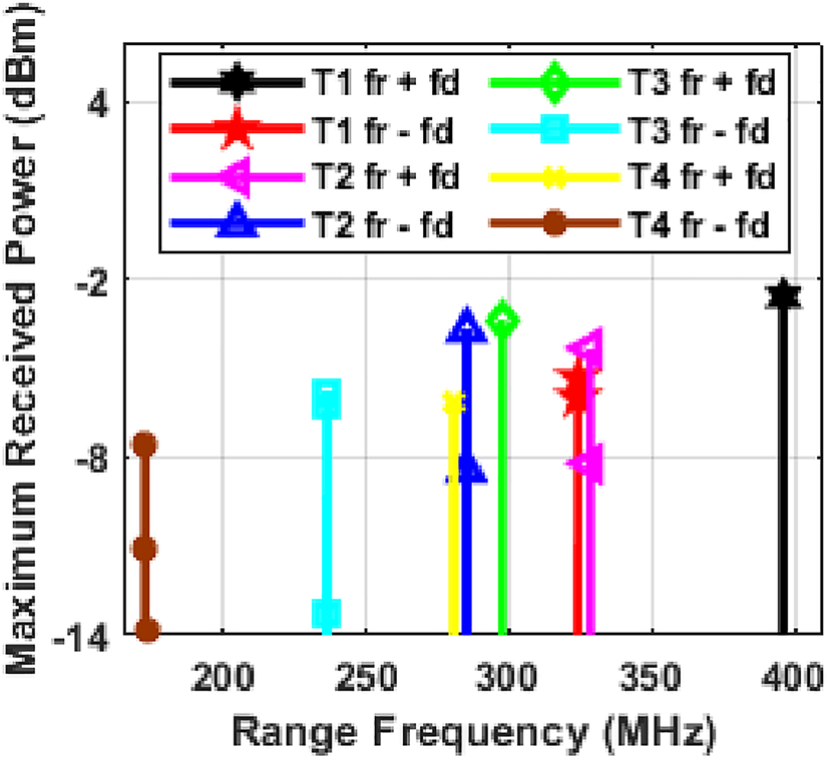
Moving target detection under Clear weather conditions and; T represents target and *f*_*r*_ + *f*_*d*_ represents vehicle moving away from the photonic radar equipped vehicle and *f*_*r*_*—f*_*d*_ represents vehicle moving towards the photonic radar equipped vehicle.

Our system's performance with moving targets is shown in Fig. 4. We assigned specific speeds to each target: 100 km/h for Target 1, 60 km/h for Target 2, 85 km/h for Target 3, and 150 km/h for Target 4. The Doppler frequencies were 35.84 MHz for Target 1, 21.50 MHz for Target 2, 30.47 MHz for Target 3, and 53.76 MHz for Target 4. The Doppler shift depends on the target's direction of movement. If a target moves towards the radar, the new range frequency equals the original minus the Doppler frequency. If moving away, it's the original plus the Doppler frequency. This method helps track target movement and adjust the range frequency accurately.

**Figure 5:**
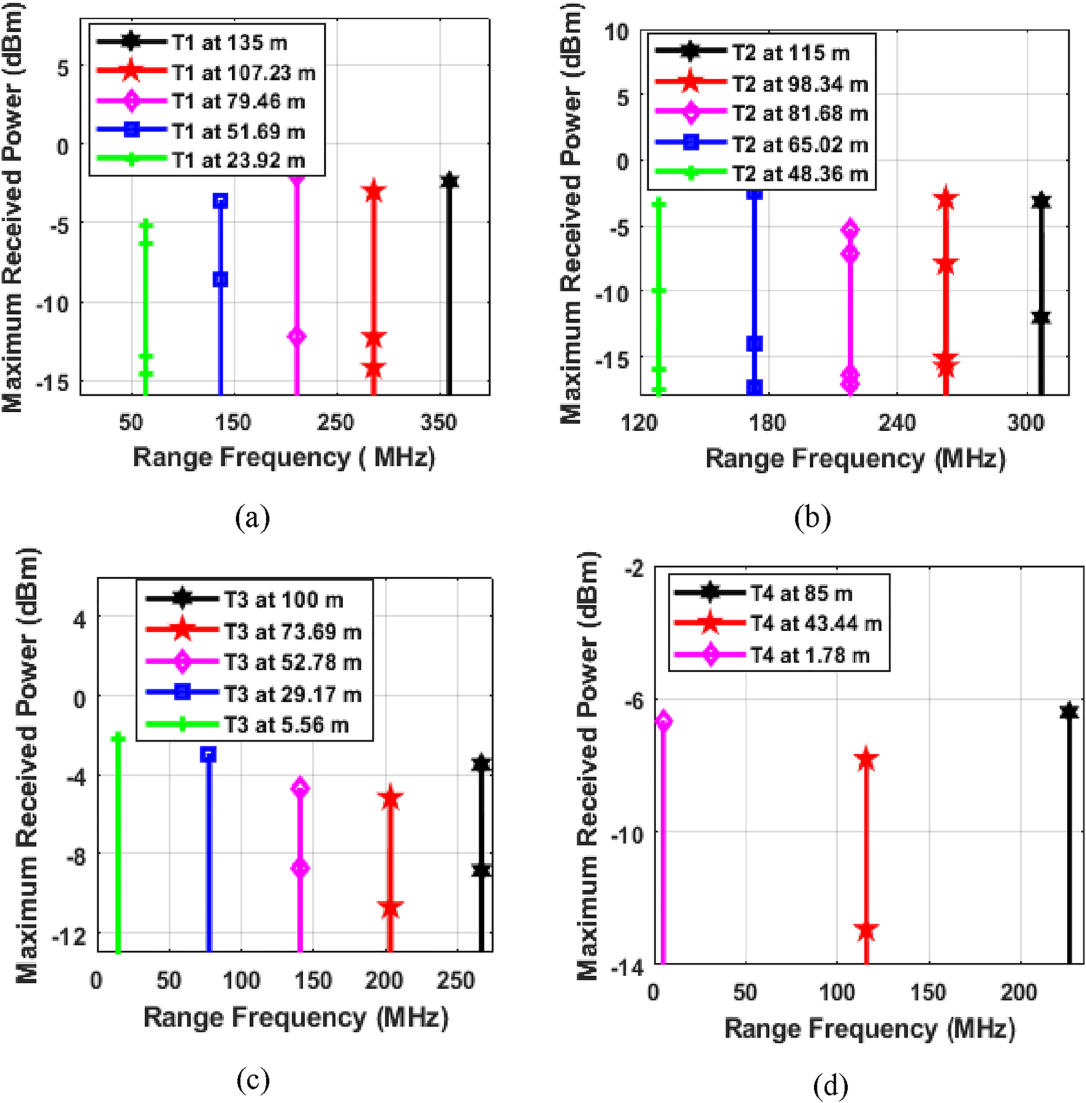
Moving target detection (**a**) Target 1 at 100 km/h, (**b**) Target 2 at 60 km/h, (**c**) Target 3 at 85 km/h and (**d**) Target 4 at 150 km/h; T represents target.

The system underwent trials for identifying moving targets at various speeds and distances. These tests included situations where the targets were either approaching or receding from the radar, as shown in Fig. 4. In every scenario, we compared the observed range frequency peaks with values calculated using Eq. (8). For instance, when Target 1 was moving away, its peak frequency *(fr* + *fd)* was noted at 395.84 MHz (marked in black in Fig. 5). In contrast, when it was approaching the radar, the peak frequency *(fr—fd)* appeared at 324.16 MHz (marked in red in Fig. 5). These observed frequencies matched the values predicted by the mathematical model, confirming the system's ability to detect and track moving targets accurately. Similarly, for Target 2, the peak frequencies observed when moving away and towards the radar were 328.16 MHz and 285.16 MHz, highlighted in pink and blue, respectively. For Target 3, these frequencies were 297.13 MHz (moving away) and 236.19 MHz (approaching), marked in green and cyan in Fig. 5. And for Target 4, the observed peak frequencies were 280.52 MHz (moving away) and 172.90 MHz (approaching), indicated in yellow and brown. These results further demonstrate the system's capability in accurately detecting and monitoring the movement of different targets under varying conditions. The findings from our simulations show that the photonic radar system we designed can successfully detect and accurately measure the distance and speed of moving targets. Further numerical simulation is being carried out on the proposed photonic radar system to improve its ability to detect potential collisions and generate warning signals based on the speed of each target.

Figure 5 is used to plot the positions of targets over time and evaluate how the speed of each target affects its position. This approach can help identify any issues with the system's ability to detect fast-moving targets and generate timely warning signals to prevent collisions. The first target in the scenario is positioned at an initial distance of 135 m from the photonic radar-equipped vehicle. This target is traveling at a relative speed of 100 km per hour in relation to the vehicle. As the vehicle moves towards the radar at a rate of 27.77 m/s, Eq. (8) is used to calculate the corresponding range frequency for the target's position every second. After one second, the target is at a distance of 107.23 m from the radar, with a corresponding range frequency of 285.946 MHz. After 2 s, the target has moved to a distance of 79.46 m, with a corresponding range frequency of 211.893 MHz. Similarly, after three seconds, the target has moved to 51.69 m, with a corresponding range frequency of 137.84 MHz, and after four seconds, it has moved to 23.92 m, with a corresponding range frequency of 63.786 MHz. These range frequencies for the first target are plotted in Fig. 5a, demonstrating the effectiveness of the proposed photonic radar system in complex conditions.

The second target is initially located 115 m away from the photonic radar-equipped vehicle, traveling at a speed of 60 km/h. As the vehicle moves towards the radar at a rate of 16.66 m/s, Eq. (8) is used to calculate the corresponding range frequency for the target's position every second. After one second, the target is at a distance of 98.34 m from the radar, with a corresponding range frequency of 262.24 MHz. After two seconds, the target has moved to a distance of 81.68 m, with a corresponding range frequency of 217.81 MHz. Similarly, after three seconds, the target has moved to 65.02 m, with a corresponding range frequency of 173.386 MHz, and after four seconds, it has moved to 48.36 m, with a corresponding range frequency of 128.96 MHz. These range frequencies for the second target are plotted in Fig. 5b, demonstrating the effectiveness of the proposed photonic radar system in complex conditions.

The third target is initially located 100 m away from the photonic radar-equipped vehicle, traveling at a speed of 85 km/h. As the vehicle moves towards the radar at a rate of 23.61 m/s, Eq. (8) is used to calculate the corresponding range frequency for the target's position every second. After one second, the target is at a distance of 73.69 m from the radar, with a corresponding range frequency of 203.706 MHz. After two seconds, the target has moved to a distance of 52.78 m, with a corresponding range frequency of 140.746 MHz. Similarly, after three seconds, the target has moved to 29.17 m, with a corresponding range frequency of 77.786 MHz, and after four seconds, it has moved to 5.56 m, with a corresponding range frequency of 14.286 MHz. These range frequencies for the third target are plotted in Fig. 5c, demonstrating the effectiveness of the proposed photonic radar system in complex conditions.

The fourth target is initially located 85 m away from the photonic radar-equipped vehicle, traveling at a speed of 150 km/h. As the vehicle moves towards the radar at a rate of 41.66 m/s, Eq. (8) is used to calculate the corresponding range frequency for the target's position every second. After one second, the target is at a distance of 43.44 m from the radar, with a corresponding range frequency of 115.84 MHz. After two seconds, the target has moved to a distance of 1.78 m, with a corresponding range frequency of 4.746 MHz. These range frequencies for the fourth target are plotted in Fig. 5d, demonstrating the effectiveness of the proposed photonic radar system in complex conditions.

Further the reducing distance of the target from the photonic radar vehicle can be used to generate warning signal such as collision alert as distance remains less than 50 m and apply the brakes when the distance remains less than 25 m.

**Figure 6:**
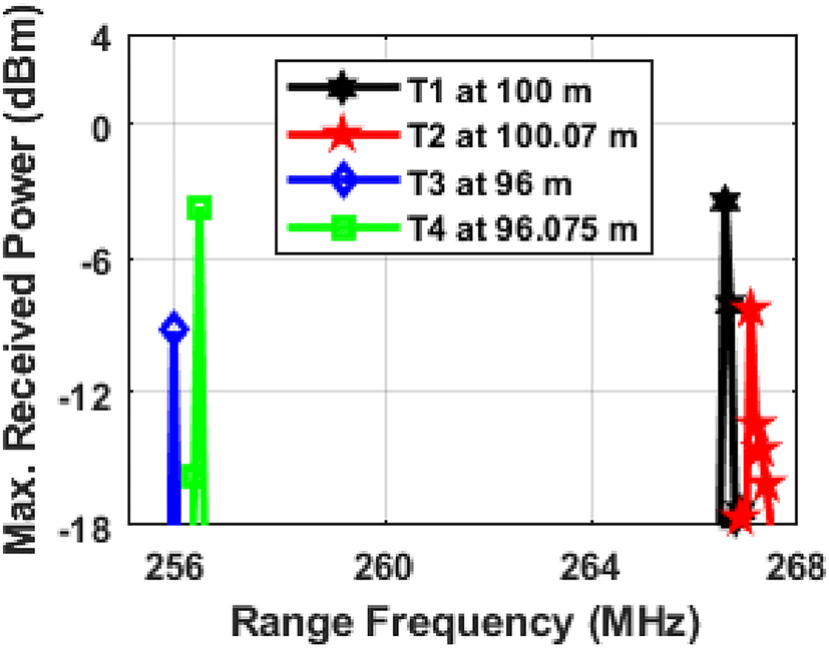
Range Resolution of proposed system.

Lastly the proposed system is tested for the range resolution. Range resolution is a measure of the photonic radar's ability to distinguish between different radar targets at different distances and is represented by the variable L_RES_ in Eq. (9) as^41^:

9 $$ L_{RES} = \frac{c}{2B} $$

where *c* is speed of light and* B* is operational bandwidth of the system. Figure 6 depicts the ability of proposed system to detect multiple closely spaced targets.

Using a 4 GHz bandwidth, the theoretical range resolution of our system is calculated to be 3.75 cm. Figure 6 shows how bandwidth affects range resolution. We positioned the first two targets (Target 1 and Target 2) 100 m and 100.07 m away, respectively, which results in a 7 cm gap between them. Similarly, the next two targets (Target 3 and Target 4) are placed 96 m and 96.075 m away, making a 7.5 cm gap between them. Our numerical simulations revealed that at a 4 GHz bandwidth, the system could detect targets with a range resolution of 7 cm, but with a 3.25 cm error. Lastly the system is trained using machine learning algorithm to classify among different targets under complex conditions such as multiple targets with and without attenuation and moving targets.

## Support vector machine design

**Figure 7:**
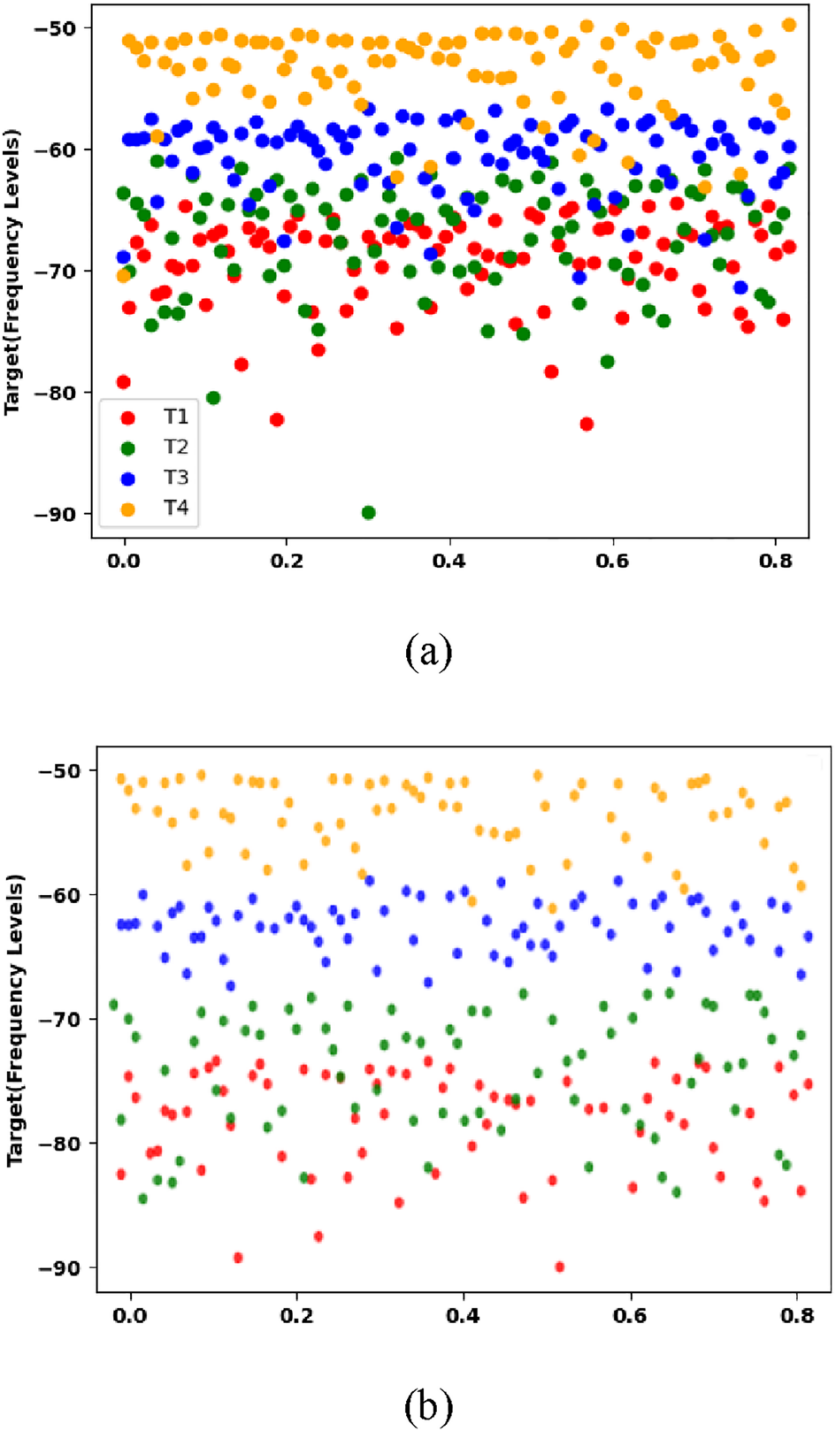
Stationary targets (**a**) actual data (**b**) SVM classified data.

In this work, we have developed machine learning algorithms that can effectively classify different targets under complex conditions, such as scenarios involving multiple targets with and without attenuation, as well as moving targets. To tackle the challenge of distinguishing between moving and stationary targets, we employed two separate SVM models. To achieve optimal classification results, we utilized the widely adopted "Radial Basis Function" (RBF) kernel, which is known for its ability to handle a large number of features. The RBF kernel has two crucial parameters: gamma and C, also referred to as the regularization parameter. In our experimental setup, we set the gamma value to 0.0542 for first SVM which has 4 target and 0.03587654 for second SVM which has 8 targets. The gamma parameter plays a significant role in determining the kernel width and has a notable impact on the accuracy of the model. It is essential to carefully choose the gamma value to avoid over-fitting or under-fitting. When gamma is set too high, over-fitting occurs, resulting in lower accuracy. On the other hand, when gamma is set too low, under-fitting occurs, leading to higher accuracy but potentially sacrificing predictive power. Therefore, finding the right value for gamma is crucial in accurately predicting the state of the target. The cost parameter C is set to 1 which controls training error and margin. In our experiments, we trained the stationary target model using 4 stationary targets located at varying distances, each has 4096 frequency samples. Figure 7 displays the classification results of the stationary targets under the effect of attenuation.

**Figure 8:**
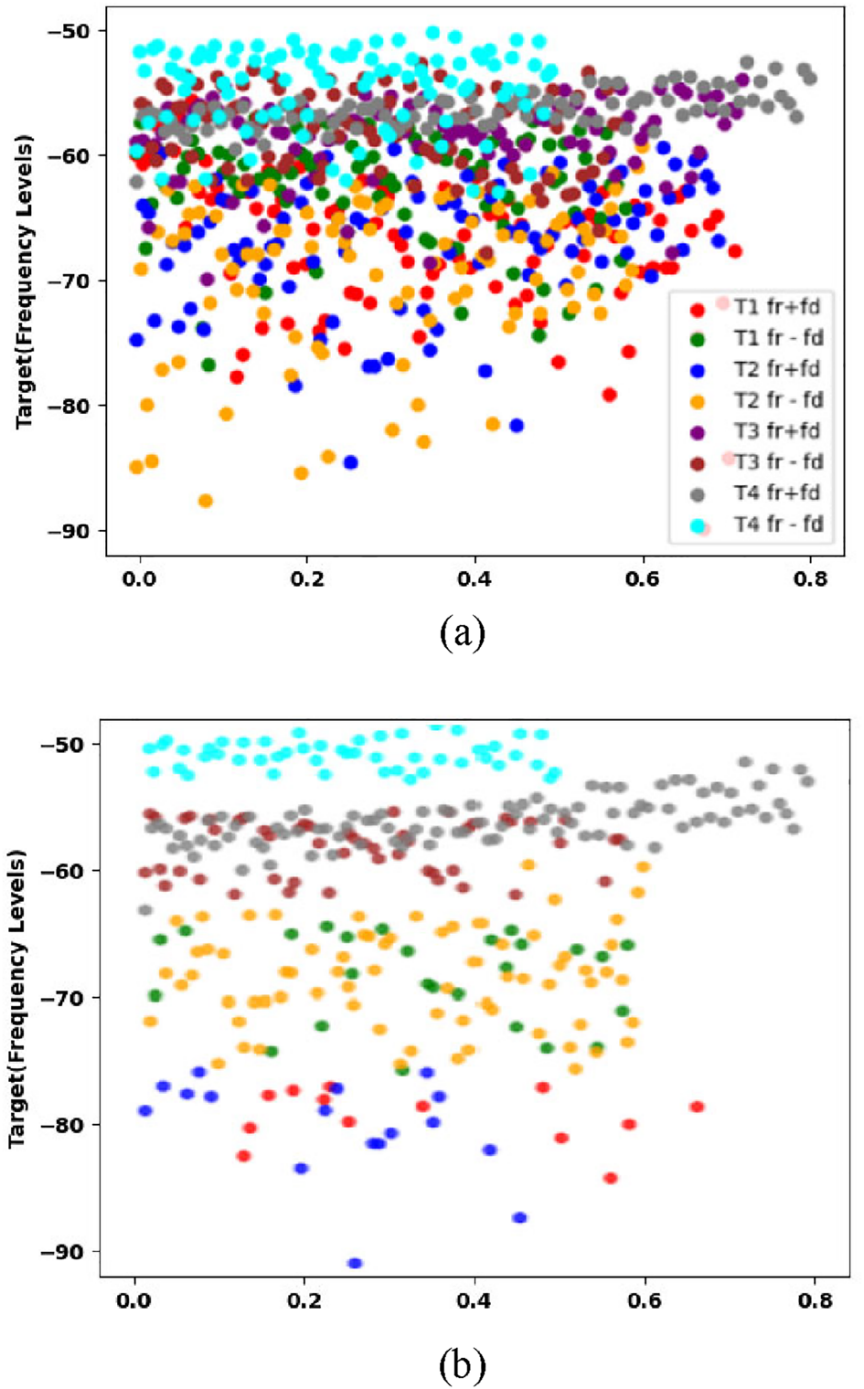
Stationary targets (**a**) actual data (**b**) SVM classified data.

Figure 7 consists of two subfigures. In Fig. 7a, the actual overlapped data for stationary targets is depicted. This means that the targets in the dataset were stationary, but due to certain conditions, their data overlapped, making it challenging to distinguish between them visually. Figure 7b shows the classified data using the developed model. The overall accuracy of the model in classifying the targets is reported to be 74%. The model successfully identifies targets T3 and T4 correctly. However, the accuracy of the model is affected by the presence of overlapping data from targets T1 and T2. This indicates that the model faces difficulties in accurately distinguishing between these two targets, which share similar characteristics. Moving forward, the moving target model was trained on four targets that exhibited movement in 8 different situations. These situations involved targets moving towards (*f*_*r*_ + *f*_*d*_) and away (*f*_*r*_*—f*_*d*_) from the system. Each target in the dataset consisted of 4096 frequency samples. Figure 8 shows the classification of moving targets.

In Fig. 8, we observe the classification of moving targets. Figure 8a presents the actual overlapped data for the stationary targets. This means that, despite the targets being in motion, their data overlaps due to certain conditions, making it difficult to visually differentiate them. Figure 8b demonstrates the classified data using the SVM model. However, in this case, the accuracy of the model is reported to be 33.23%. This means that the model correctly classified only a third of the targets in the dataset with a huge amount of overlapped dataset. The performance of the SVM model is adversely affected by the presence of overlapping data from multiple targets. Even when some targets are separated by other targets, the issue of overlapping similar targets persists, making it challenging for the model to accurately distinguish between them.

## Conclusion

In this work, we proposed a photonic radar system for autonomous vehicle applications. The system utilizes linear frequency-modulated continuous wave and mode division multiplexing in a heterodyne configuration. It enables the detection and precise measurement of target range in both stationary and moving positions using a coherent detection method. Our system has several benefits, such as low power needs, being small in size, and being easy to set up. The proposed photonic radar successfully spotted four targets that were 135 m, 115 m, 100 m, and 85 m away from a vehicle with the radar. We confirmed these findings using standard math formulas. We also tried out the system with moving targets, which were going at speeds of 100 km/h, 60 km/h, 85 km/h, and 150 km/h. For these tests, we figured out the Doppler effect and checked the distance frequencies. Additionally, we looked at how well the system could tell distances apart by using it with a bandwidth of 4 GHz. The reported results showed range resolutions of 7 cm with an error of 3.25 m from theoretically calculated value. Final Machine learning methods are used to classify different targets under stationary and moving target conditions using SVM algorithm. An accuracy of 74% is obtained in the classification of the targets. Future research will focus on several key areas to further enhance the capabilities of the proposed system. One potential direction is the integration of deep learning algorithms, such as convolutional neural networks (CNNs) and recurrent neural networks (RNNs), to improve classification accuracy. These methods can handle more complex patterns and provide better performance in distinguishing between multiple moving targets in dense traffic scenarios. Additionally, exploring other machine learning algorithms, such as Random Forest, Gradient Boosting, and Ensemble Learning, could offer improved accuracy and robustness in target detection and classification.

Another promising area for future research is the fusion of multiple radar modalities. Combining photonic radar with traditional microwave radar or LiDAR could enhance the system's ability to detect and classify targets in diverse environmental conditions. Furthermore, developing adaptive signal processing techniques to dynamically adjust the system parameters based on the traffic scenario and environmental conditions could improve the system's performance in real-time applications.

Integrating the photonic radar system with vehicle-to-everything (V2X) communication systems can provide a comprehensive understanding of the traffic environment, leading to more accurate and reliable target detection and classification. Conducting extensive field testing and validation in various real-world traffic scenarios will be crucial to assess the system's performance and identify areas for further improvement. Thus, the proposed photonic radar system demonstrates significant potential for enhancing autonomous vehicle navigation. Future research will focus on integrating advanced machine learning techniques, exploring different radar modalities, and conducting extensive field testing to further optimize the system's performance and reliability in complex traffic environments.

## Data Availability

There is no data available to declare. All the data is mentioned within the manuscript.
